# Distribution of diabetic retinopathy in diabetes mellitus patients and its association rules with other eye diseases

**DOI:** 10.1038/s41598-021-96438-w

**Published:** 2021-08-20

**Authors:** Xi Yao, Xiaoting Pei, Yingrui Yang, Hongmei Zhang, Mengting Xia, Ranran Huang, Yuming Wang, Zhijie Li

**Affiliations:** 1 Henan Eye Institute, Henan Eye Hospital, and Henan Key Laboratory of Ophthalmology and Visual Science, Henan Provincial People’s Hospital, People’s Hospital of Zhengzhou University, People’s Hospital of Henan University, Zhengzhou City, 450000 China; 2 Nursing Department, Henan Provincial People’s Hospital, People’s Hospital of Zhengzhou University, People’s Hospital of Henan University, Zhengzhou, China; 3 Departments of Science and Technology Administration, Henan Provincial People’s Hospital, Henan University People’s Hospital, Zhengzhou University People’s Hospital, Zhengzhou, China

**Keywords:** Diseases, Health care, Medical research

## Abstract

The study aims to explore the distribution characteristics and influencing factors of diabetic retinopathy (DR) in diabetes mellitus (DM) patients and association rules of eye diseases in these patients. Data were obtained from 1284 DM patients at Henan Provincial People’s Hospital. Association rules were employed to calculate the probability of the common occurrence of eye-related diseases in DM patients. A web visualization network diagram was used to display the association rules of the eye-related diseases in DM patients. DR prevalence in people aged < 40 years (≥ 58.5%) was higher than that in those aged 50–60 years (≤ 43.7%). Patients with DM in rural areas were more likely to have DR than those in urban areas (56.2% vs. 35.6%, *P* < 0.001). DR prevalence in Pingdingshan City (68.4%) was significantly higher than in other cities. The prevalence of DR in patients who had DM for ≥ 5 years was higher than in other groups (*P* < 0.001). About 33.07% of DM patients had both diabetic maculopathy and DR, and 36.02% had both diabetic maculopathy and cataracts. The number of strong rules in patients ≥ 60 years old was more than those in people under 60 in age, and those in rural areas had more strong rules than those in urban areas. DM patients with one or more eye diseases are at higher risks of other eye diseases than general DM patients. These association rules are affected by factors such as age, region, disease duration, and DR severity.

## Introduction

According to data from the International Diabetes Federation (IDF), there were 463 million diabetes mellitus (DM) patients worldwide in 2019. This number will probably grow to around 700 million by 2045^1^. Diabetic retinopathy (DR) is a retinal disease secondary to damage to retinal capillaries caused by impaired glucose tolerance, and is recognized as an inflammatory neurovascular complication with neurological impairment/dysfunction^2^, which eventually severely affects vision and even results in blindness^3^. Therefore, DR is one of the most common serious complications of DM^4,5^. The reported prevalence of DR in type 2 diabetes patients in developing and developed countries ranges from 12.2 to 61.0% and 9.9 to 48.1%, respectively^6^. Given the high prevalence of DR, high possibility of blindness, and low levels of awareness about DR, this condition severely reduces the quality of life of patients and has become a major public health problem.

As a result of economic development and social progress, as we have come to understand, the occurrence of diseases is sometimes not limited to the traditional scenarios of one cause, one disease; one cause, multiple diseases; and multiple causes, one disease. Research has shown that the study of multi-cause and multi-effect pathogeneses may better reflect the nature of diseases. In addition, eye-related diseases are not independent of each other; they have complex relationships. Studies have also revealed that the presence of one disease increases the risk of the occurrence of second and even third diseases^7^. For example, the probability of cataract occurrence in glaucoma patients is much higher than that in the general population^8^. Therefore, studying the distribution of eye-related diseases and the association rules among them may be helpful for the early detection and treatment of eye diseases, including DR.

Therefore, we collected clinical data from DM patients who were examined in the Department of Ophthalmology at Henan Provincial People’s Hospital from 2018/08 to 2020/06. Multiple correspondence analysis and association rules were used to analyze the distribution characteristics of DR and determine its association rules with other eye diseases in DM patients. These data provided a frame of reference for developing appropriate measures for better screening, diagnosis, and prevention of DR.

## Methods

### Study participants

The data in this cross-sectional study were obtained from the Hospital Information System of Henan Provincial People’s Hospital. DM patients treated at the Fundus Disease Center of Henan Provincial People’s Hospital from 2018/08/01 to 2020/06/10 who were ≥ 18 years old were included in the study. The exclusion criteria were as follows: patients with missing key variables (such as DR diagnosis results) or who were missing > 30% of the variables; individuals with missing fundus images; those with failure of the heart, liver, kidney, or another important organ, as the circulatory system and medication of these patients may have an impact on the diagnosis and treatment of DR; and patients with malignant tumors. Demographic characteristics (age, gender, hometown), diagnoses (DR, cataracts, glaucoma, age-related macular degeneration, diabetic maculopathy and refractive errors, hypertension), fundus photographs and duration of disease were independently collected by two authors and checked for consistency between these two authors. The study was approved by the Ethics Committee of Henan Provincial People’s Hospital, and written informed consent was obtained from all participants. All methods were performed in accordance with the relevant guidelines and regulations.

### Definitions and diagnostic criteria

DM was diagnosed according to the Standards of Medical Care in Diabetes established by the American Diabetes Association^9^. Fundus photographs of each participant were taken according to unified standards by an ophthalmologist using a Zeiss non-mydriatic fundus camera (VISUCAM 224, Germany), with the macula as the center and 45° Color mode shooting. Five fields were captured in each eye: macula center, temporal side, nasal side, upper and lower quadrants of the retina. Then, we selected the clearest picture from the three photographs as the final fundus photograph of the patient.

DR diagnosis and grading were conducted by two clinicians (with ≥ 5 years of experience) based on international clinical diagnosis and classification standards^10^. DR was divided into five stages: (0) absence of DR (NDR), (1) mild non-proliferative diabetic retinopathy (NPDR), (2) moderate NPDR, (3) severe NPDR and (4) proliferative diabetic retinopathy (PDR). When the grading results of the two clinicians were not in agreement, adjudication by a panel of four retina specialists served as the reference standard. Patients with DR in both eyes were regarded as one case and included in the analysis. Those with different degrees in each eye were graded according to the more severe eye. Cataracts^11^, glaucoma^12^, age-related macular degeneration^13^, diabetic maculopathy^14^ and refractive errors^15^ were diagnosed by ophthalmologists according to standard clinical diagnostic criteria. Microaneurysm^16^, optic atrophy^17^ and tessellated retina^18^ were determined based on the fundus photographs and references.

Vision was measured using the standard logarithmic visual acuity chart with the participants standing 5 m in front of the chart. The smallest line of vision that could be recognized by the examined eye was recorded as the value of the vision, which is the visual acuity of the examined eye. The vision of the eye with better vision was considered to be the participant's vision for the final analysis. The intraocular pressure of the eyes was measured using a non-contact tonometer (Canon TX-20P) according to the manufacturer’s guidelines. The average of three measurements of each eye was taken as the value for each eye. Bergmeister papilla was observed using optical coherence tomography (OCT; Carl Zeiss Meditec, Inc.) focused on the optic cup and optic disc. The vertical and horizontal diameters of the optic cup and optic disc were measured using the measuring tool of the OCT machine. The average diameter of the optic cup or optic disc was calculated as the sum of the vertical and horizontal diameters divided by two.

### Statistical analyses

IBM SPSS Statistics 23.0 and SPSS Modeler 18.0 software (SPSS Inc., Chicago, IL) were used for the statistical analysis. For the qualitative variables, frequency was descripted. The *χ*^2^ test was used for comparisons between groups. For the quantitative variables, mean ± standard deviation (SD) was determined, and t-tests were used for between-group comparisons. To observe the age distribution of DR, most of the patients were divided into seven age groups, spanning 10 years each, except for the first group, which included individuals aged 18.0–29.9 years. Those ≥ 80 years were merged into one group due to the small sample size. Regional distribution was created using free online software (http://c.dituhui.com/). Multiple correspondence analysis was employed to show the relative relationship of eye-related diseases in DM patients. Apriori algorithm of association rules was used to determine the strength of the rule through the support (S) and the confidence (C)^19^, which were used to calculate the probability of co-occurrence of eye-related diseases in DM patients. Conditional support was given as S(X) = P(X), where P is the probability of the occurrence of X; and rule support S(X ⟹ Y) = P(X ∪ Y) is the probability of X and Y occurring at the same time. In this study, X and Y refer to eye-related diseases. Confidence, C(X ⟹ Y) = P(X⁄Y) = S(X ⟹ Y)/S(X), is the conditional probability of Y occurring under the condition that X occurs. Lift, P(Y|X)/P(X), reflects the correlation between X and Y in the association rules. Lift > 1 indicates a higher positive correlation; lift < 1 indicates a higher negative correlation; and lift = 1 indicates no correlation. In this study the minimum support, confidence, and lift were set to 15%, 80%, and 1.10, respectively. A web visualization network diagram was employed to show the association rules among the eye-related diseases of DM patients. All the P-values were two-tailed, and the level of significance was set at α = 0.05.

### Ethics approval and consent to participate

The protocol for the study was approved by the Ethics Committee of Henan Provincial People’s Hospital, and written consent was obtained from all the participants.

## Results

### Participants’ general characteristics

A total of 1300 DM patients aged 18–97 years were included in this study, in which, 16 patients were excluded due to the poor quality of images. Finally, 1284 DM patients were used for the analysis. There were 628 (48.9%) males and 656 (51.1%) females, with an average age of 62.23 ± 12.00 years and an average duration of DM of 10.00 (3.00–15.00) years. The average visual acuity was 0.55 ± 0.36. Among these patients, 580 (45.2%) had DR, 754 (58.7%) suffered from cataracts, 152 (11.8%) had glaucoma, and 422 (32.9%) had hypertension. The general characteristics of the DM patients are shown in Table 1.

**Table 1:** General characteristics of the DM patients.

	DR (n = 580)	NDR (n = 704)	*t*/*χ*^2^	*P*
Age (years old)	58.11 ± 10.91	65.62 ± 11.79	11.737	< 0.001
Ratio of optic cup to optic disc	0.49 ± 0.23	0.45 ± 0.17	3.618	< 0.001
Vision	0.52 ± 0.36	0.58 ± 0.36	2.631	0.009
Intraocular pressure of right eye (mmHg)	17.58 ± 6.75	17.19 ± 5.23	1.029	0.304
Intraocular pressure of left eye (mmHg)	17.24 ± 6.46	17.31 ± 4.50	0.192	0.847
Optic disc (mm)	1.83 ± 0.17	1.85 ± 0.20	1.937	0.053
Optic cup (mm)	0.75 ± 0.22	0.71 ± 0.18	3.515	< 0.001
Gender (male/female)	280/300	348/356	0.170	0.680
Cataracts (yes/no)	280/300	474/230	47.630	< 0.001
Refractive error (yes/no)	34/546	184/520	92.735	< 0.001
Glaucoma (yes/no)	72/508	80/624	0.336	0.562
Hypertension (yes/no)	134/446	288/416	46.694	< 0.001

### Distribution characteristics of DR in DM patients

**Figure 1:**
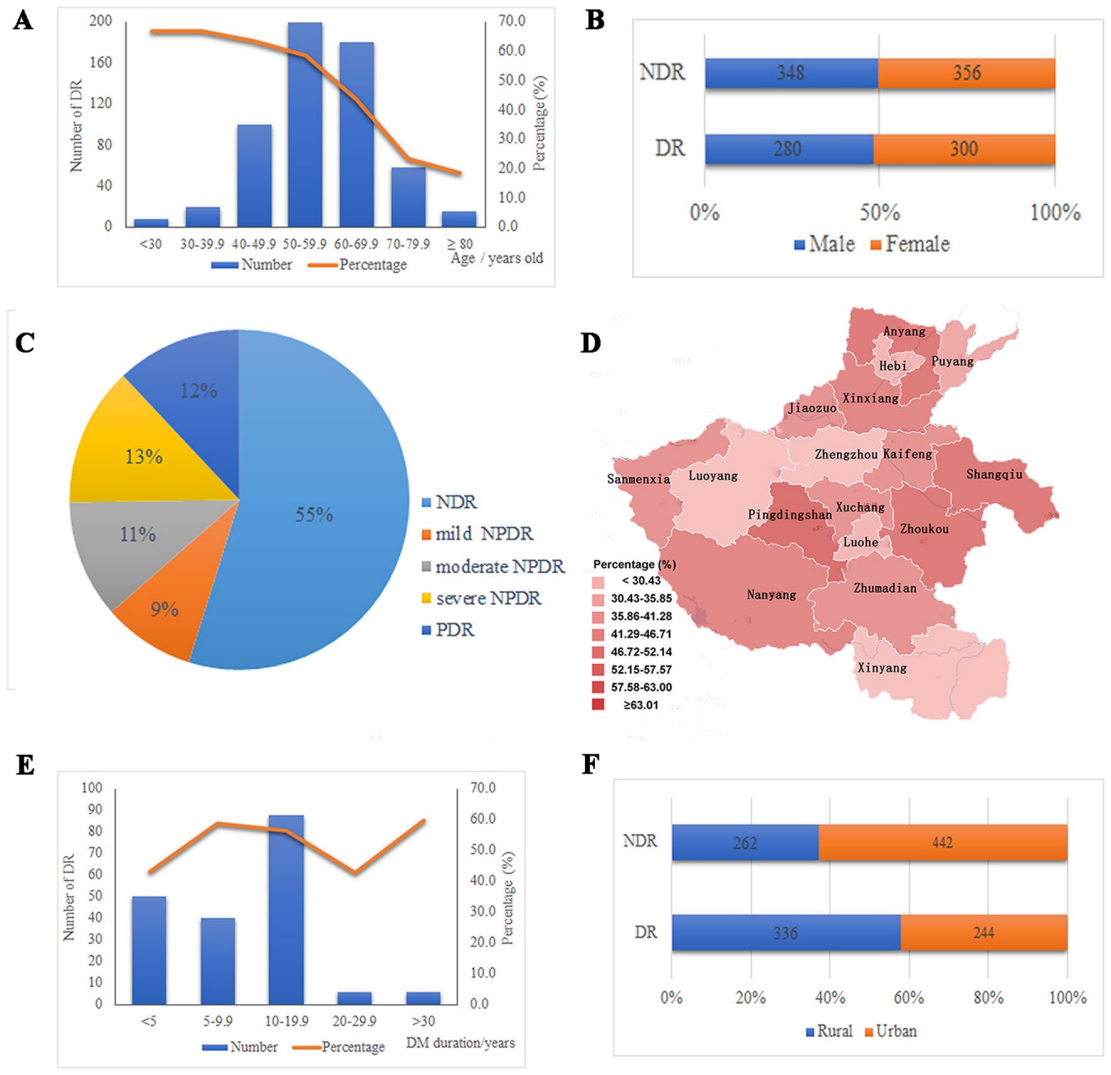
Distribution characteristics of DR in DM patients. (**A**) Age distribution of DR in DM patients; (**B**) Gender distribution of DR in DM patients; (**C**) Distribution of DR according to severity levels; (**D**) Regional distribution of DR in DM patients; (**E**) Relationship between DR and the disease duration of DM; (**F**) Number of DR cases in rural and urban patients.

While there were more DR patients in the 50–60 age group, the prevalence of DM in the population under 40 years old was higher than that in the 50–60 age group (58.5–66.7%) (Fig. 1A). There was no statistically significant difference in the prevalence of DR between males and females with DM (*χ*^2^ = 1.777, *P* = 0.680) (Fig. 1B). Among the 580 DR patients, 112 had mild NPDR, 144 had moderate NPDR, 170 had severe NPDR and 154 had PDR (Fig. 1C). DM patients in Pingdingshan (68.4%), Shangqiu (62.5%), Anyang (60.0%), and Zhoukou (58.2%) cities had a higher risk of DR (*χ*^2^ = 43.582, *P* < 0.001) than those from other areas (Fig. 1D). There was a higher risk of DR in DM patients with a disease duration of > 5 years (42.9–60.0%) (*χ*^2^ = 114.219, *P* < 0.001) than those with a disease duration of ≤ 5 years (Fig. 1E). There was a statistically significant difference in the prevalence of DR between rural and urban patients (*χ*^2^ = 57.199, *P* < 0.001) (Fig. 1F).

### Multiple correspondence analysis

**Figure 2:**
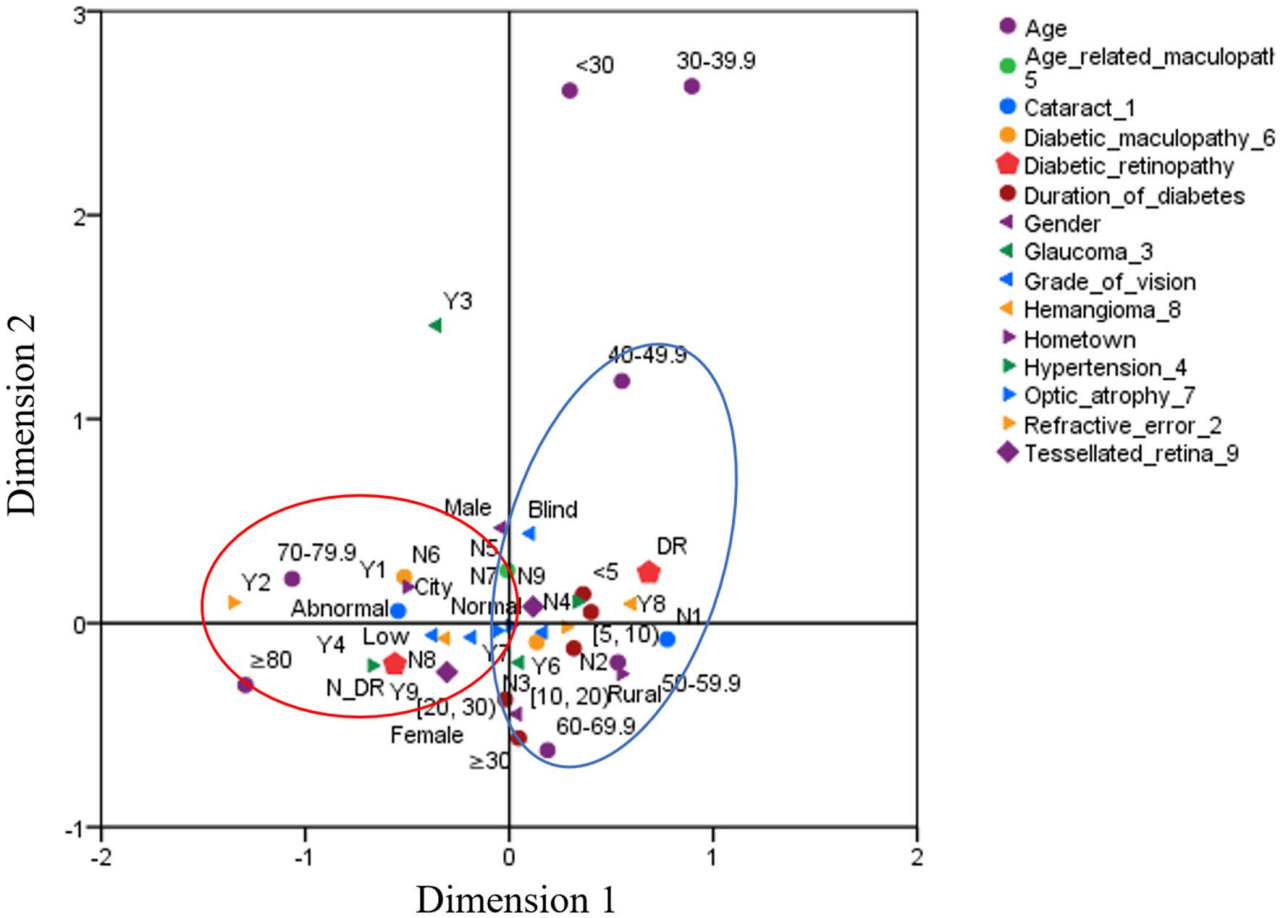
Multiple correspondence analysis of population characteristics and eye-related diseases.

Multiple correspondence analysis was performed to intuitively understand the relationship between eye-related diseases and DR in DM patients, in which, age, gender, DM duration, hometown, cataracts, glaucoma, age-related maculopathy, diabetic maculopathy, hypertension, optic atrophy, and DR were employed as analysis variables. The results showed that glaucoma, age-related macular disease, refractive error, and optic atrophy had no obvious aggregation phenomenon from the joint plot of the category points, while the attributes of being a rural patient, blindness, hemangioma, and an age ≥ 40 years had obvious aggregations with DR (Fig. 2).

### Association rules of eye diseases in DM patients

**Figure 3:**
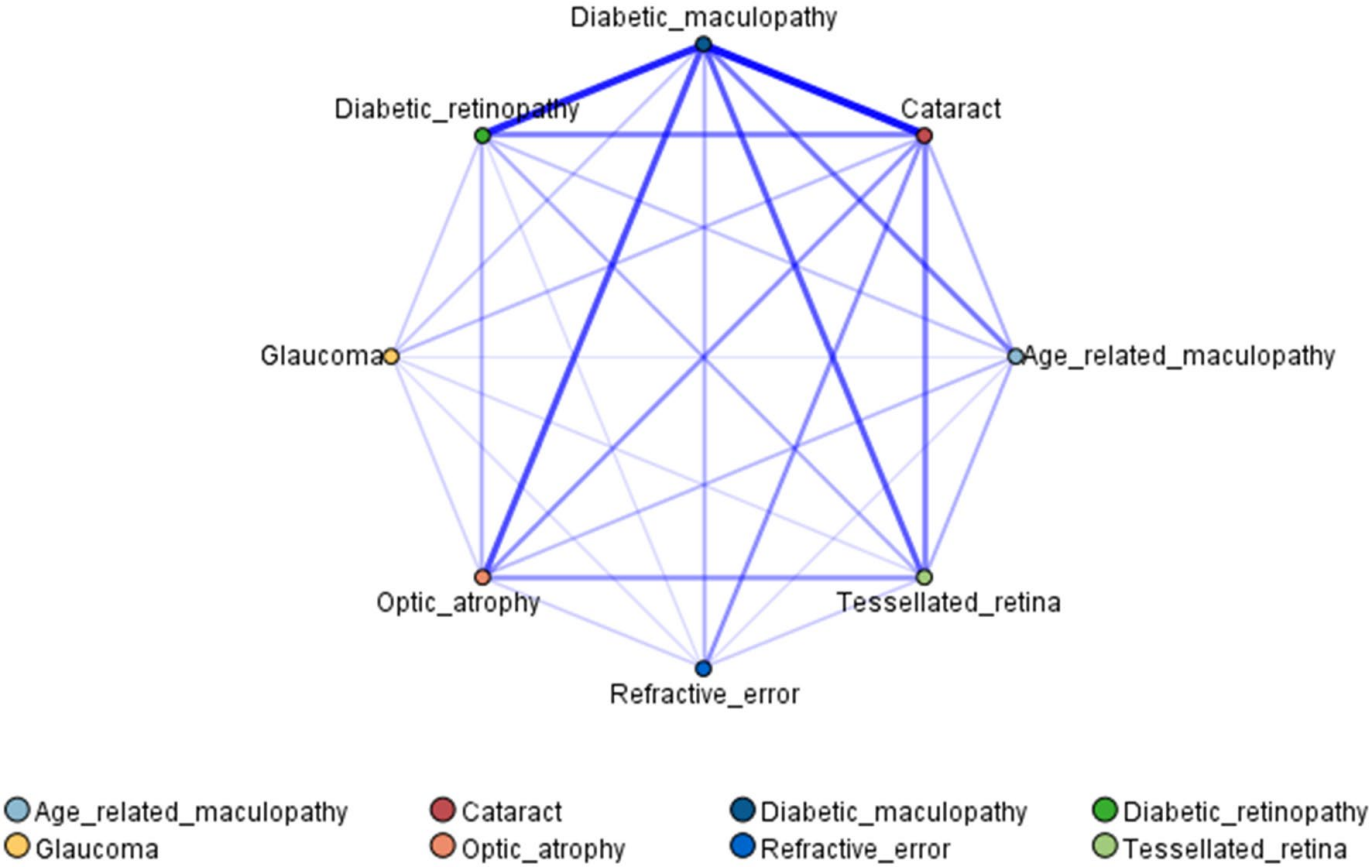
Web visualization network diagram of eye diseases in DM patients.

**Figure 4:**
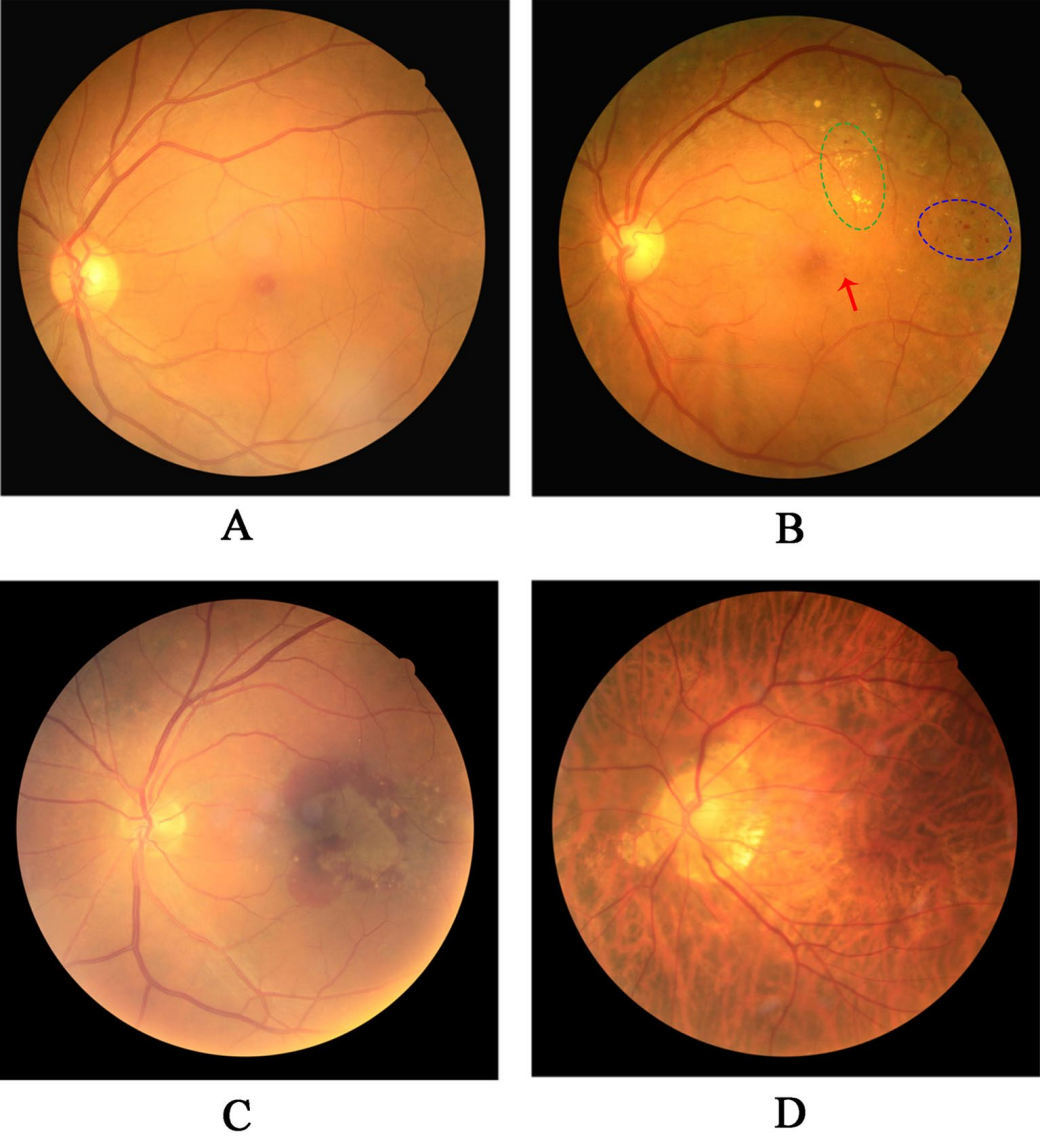
Typical fundus photographs in patients with DM. (**A**) Fundus photograph of a healthy individual; (**B**) Fundus photograph of a DR patient (blue circle indicates retinal hemorrhage, green circle indicates hard exudation, and red arrow indicates microaneurysms); (**C**) Fundus photograph of an age-related macular disease patient; (**D**) Fundus photograph of a tessellated retina patient.

A web visualization network was developed to visually observe the association rules among eye-related diseases in DM patients (Fig. 3). Typical fundus photographs in patients are shown in Fig. 4. The results showed that of the DM patients, 426 (33.07%) had both diabetic maculopathy and DR, 464 (36.02%) had both diabetic maculopathy and cataracts, 326 (25.31%) had both diabetic maculopathy and optic atrophy. There were 298 (23.14%) DM patients with both tessellated retinas and diabetic maculopathy, 238 (18.48%) had both DR and cataracts.

Among the 1284 DM patients included, 1278 (99.5%) had eye diseases. Results of association rules showed that the co-occurrence of DR, diabetic macular degeneration, and cataracts among the DM patients was very high, and the lift was greater than 1.1, indicating that the prevalence of eye diseases was positively correlated (Table 2).

**Table 2:** A priori algorithm of association rules.

Group	Latter term	Former term	Support %	Confidence %	Lift
**Total**	Diabetic maculopathy	Age-related maculopathy	20.51	95.87	1.25
	Diabetic maculopathy	Optic atrophy	30.34	91.06	1.18
	Cataract	Refractive error	17.97	90.57	1.55
	Diabetic maculopathy	Optic atrophy, tessellated retina	16.27	87.50	1.14
**Age**					
< 40	Diabetic retinopathy	Tessellated retina	25.00	100.00	1.54
	Diabetic maculopathy	Tessellated retina, diabetic retinopathy	25.00	100.00	1.25
	Diabetic retinopathy	Tessellated retina, diabetic maculopathy	25.00	100.00	1.54
[40,60)	Diabetic maculopathy	Age-related maculopathy	15.25	100.00	1.21
	Diabetic maculopathy	Optic atrophy, diabetic retinopathy	15.70	100.00	1.21
	Diabetic maculopathy	Optic atrophy	27.80	96.77	1.17
≥ 60	Cataract	Refractive error	22.19	97.40	1.42
	Diabetic maculopathy	Age-related maculopathy	24.78	94.19	1.29
	Diabetic maculopathy	Optic atrophy	32.28	88.39	1.21
	Diabetic maculopathy	Optic atrophy, tessellated retina	21.33	86.49	1.19
	Diabetic maculopathy	Optic atrophy, cataract	18.73	83.08	1.14
**Gender**					
Male	Diabetic maculopathy	Age-related maculopathy	17.07	97.96	1.26
	Diabetic maculopathy	Optic atrophy	33.80	90.72	1.16
	Cataract	Refractive error	17.77	90.20	1.61
Female	Diabetic maculopathy	Age-related maculopathy	23.76	94.44	1.24
	Diabetic maculopathy	Optic atrophy, tessellated retina	15.51	91.49	1.21
	Diabetic maculopathy	Optic atrophy	27.06	91.46	1.20
	Cataract	Refractive error	18.15	90.91	1.51
	Diabetic maculopathy	Optic atrophy, cataract	15.51	87.23	1.15
**Area**					
Rural	Diabetic maculopathy	Age-related maculopathy	19.49	98.15	1.31
	Diabetic maculopathy	Optic atrophy	33.57	87.10	1.16
	Diabetic maculopathy	Optic atrophy, tessellated retina	20.58	84.21	1.12
Urban	Diabetic maculopathy	Optic atrophy	26.47	96.83	1.22
	Diabetic maculopathy	Age-related maculopathy	22.69	94.44	1.19
**DM duration**					
≤ 10 years	Diabetic maculopathy	Age-related maculopathy	27.59	100.00	1.12
	Diabetic maculopathy	Age-related maculopathy, tessellated retina	16.09	100.00	1.12
> 10 years	Diabetic maculopathy	Tessellated retina	27.85	100.00	1.14
	Diabetic maculopathy	Tessellated retina, optic atrophy	20.25	100.00	1.14
	Diabetic maculopathy	Tessellated retina, cataract	15.19	100.00	1.14
	Diabetic maculopathy	Tessellated retina, diabetic retinopathy	15.19	100.00	1.14
**Stage of DR**					
DR ≤ 3	Diabetic maculopathy	Age-related maculopathy	22.90	97.96	1.26
	Diabetic maculopathy	Optic atrophy	29.21	92.00	1.18
	Cataract	Refractive error	20.33	89.66	1.54
	Diabetic maculopathy	Optic atrophy, tessellated retina	17.29	87.84	1.13
	Diabetic maculopathy	Diabetic retinopathy	34.35	87.07	1.12
DR > 3	Diabetic maculopathy	Optic atrophy, diabetic retinopathy	21.60	94.29	1.27
	Diabetic maculopathy	Optic atrophy	33.33	88.89	1.20

In patients under 40 years, the support and confidence of the rule “Tessellated retina, diabetic maculopathy → DR” were 25% and 100%, respectively, that is, 25% of those younger than 40 years had both tessellated retinas and diabetic maculopathy, and the prevalence of DR in this group was 100%, which is 1.54 times that found in the DM patients without tessellated retinas and diabetic maculopathy. The number of strong rules in patients ≥ 60 years old was more than that in those under 60 years old. The co-occurrence rate of optic atrophy and tessellated retina was 25%, and the prevalence of diabetic maculopathy in this population was 86.49%, which is 1.19 times that in the DM patients without optic atrophy and tessellated retina. In urban areas, 24.55% of patients had refractive errors, and 91.18% of this group had cataracts, which is 1.35 times the rate for the general population. Rural patients had more rules than those in urban areas. In DM patients with a disease duration of less than 10 years, there was no strong rule. In DM patients with a disease duration of more than 10 years, the probability of tessellated retina and DR occurring at the same time was 15.19%, and the prevalence of diabetic maculopathy was 100% in patients with both tessellated retinas and DR, which is 1.15 times that found in the population without tessellated retinas and DR. In addition, the results showed that the number of strong rules in male patients with DM was fewer than in female patients.

## Discussion

DR can very easily cause blindness, which seriously affects a patient’s quality of life^20^. In this study, we observed 1284 DM patients with different ocular diseases at the Henan Provincial People’s Hospital, and explored the distribution and association rules of DR in these populations using multiple correspondence analysis and association rules. The results showed that: (1) among the included DM patients, the prevalence of DR varied with ages, regions, and DM durations, but there was no statistical difference in the prevalence of DR between males and females; (2) patients with one or more eye diseases are at a higher risk of other eye diseases than those in the general population, and these association rules are affected by age, disease duration, and grade of DR classification.

The proportion of DR in DM patients aged 40–60 years was significantly higher than that in people > 60 years old. This may be due to the fact that young and middle-aged people face more stress factors from comprising the main workforce in society^21^. These factors include a fast work pace, a high level of mental stress, high levels of endocrine disorders, and increased catecholamines; these can easily trigger retinal vasoconstriction causing ischemia and hypoxia of the retina^22^. In addition, the prevalence of type 1 diabetes in young patients is higher. The study showed that the prevalence of DR in type 1 DM was higher than in patients with type 2 DM, which may be associated with the higher prevalence of diabetic retinopathy in young patients^23^. Ineffective blood glucose control in patients with DM is often accompanied by lipid metabolism disorders^24^. The combined effect of all these factors causes a reduction in barrier function of vascular endothelial cells and the ability of red blood cells to carry oxygen, which will lead to the onset of other eye diseases in DR patients^25^. Therefore, patients with DM should pay strict attention to their lifestyle and eating habits, including learning how to relieve stress, using medication responsibly, regularly checking their blood glucose and the functional status of other tissues and organs, maintaining blood glucose stability, and avoiding the further deterioration of DM.

In addition, the regional distribution of the included DM patients was observed. The results showed that DM patients in rural areas of Henan were more likely to develop DR than those in urban areas. However, a meta-analysis by Song et al.^26^ showed the prevalence of DR was higher in urban than in rural settings. This inconsistency may be because the population structure of our study is different from those of other researches. Epidemiological surveys have shown that DM patients in rural areas of Henan have a significantly lower level of cognition and self-management ability for DM than those in urban areas^27^, which can lead to an increase in the possibility and speed of progression of DM to DR. Therefore, strengthening the public health services of rural grassroots medical staff and guiding patients in effective self-management may be key in comprehensively improving the effectiveness of DR prevention and control in rural patients. It is noteworthy that in the 17 cities in Henan Province, the prevalence of DR in DM patients in Pingdingshan City was significantly higher than in patients in other regions. This may be due to the diet in this region. Studies have shown that the daily dietary fat intake of residents in this area is significantly higher than those in other areas in Henan Province; in particular, the intake of edible oil is higher than the recommended amount, and the intake of cereals and beans is lower^28^. Therefore, strengthening the self-management of the diet of patients with DM and ensuring a balanced and controlled diet is essential to reducing the incidence of DM and, consequently, the prevalence of DR^29,30^.

Consistent with the results in the literature^31,32^, this study found that the occurrence of DR was closely related to the duration of DM. In particular, there was a higher proportion of DR in patients with a diabetes duration of ≥ 5 years than those in other groups. This result is expected given the fact that the longer the duration of the disease, the longer patients are exposed to many risk factors and the higher the incidence of various DM-related chronic complications^33^, which may be caused by the interaction of multiple factors. Some studies have shown that in DM patients with good glycemic control, the occurrence and development of DR can still be observed when the disease duration is long, which may be related to the memory of blood glucose metabolism^34^. Therefore, it is necessary to regularly monitor the fundus photographs of DM patients. Regardless of whether blood glucose control is stable, the frequency of eye follow-ups should be increased with time^35^. DM-related fundus changes should be found early and treated as soon as possible to prevent the further development of the disease.

The results of multiple correspondence analyses and studies showed that macular edema, hemangioma, hard exudates and retinal hemorrhages were the most common signs of DR. Traditionally, DR is regarded as a microvascular disease, and its main pathogenic links are closely related to pathological cascades, such as retinal inflammation triggered by hyperglycemia, blood-retinal barrier destruction, neovascularization, increased vascular permeability, and bleeding. Therefore, the strict control of blood glucose concentration is a key factor in controlling the occurrence of many diseases, including DR.

Finally, we analyzed the comorbidity of the eye diseases in DM patients. The results showed that the probability of comorbidity of DR, diabetic maculopathy, and cataracts in DM patients was high with a lift > 1.1, which suggests that the prevalence of these diseases is positively correlated. In addition, we found that for the same rule, the correlation strength was different in people with different characteristics. For example, “refractive error → cataract” was a strong correlation in DM patients who were > 60 years old, lived in urban areas, and had DR grades ≤ 3, while in DM patients younger than 60 years who lived in rural areas, and had DR grades > 3, there was no such association rule. There was no association rule in patients with a diabetes duration ≤ 10 years, while there were five strong association rules in patients who had DM for > 10 years. It is noteworthy that in DM patients with a duration of ≥ 10 years and combined with tessellated retina, the prevalence of diabetic maculopathy was 100%. Tessellated fundus was recognized as one of the markers in identifying high myopia. In high myopia, with the increase of axial length, sclera becomes thinner^36^, and the retina and choroid are mechanically stretched, which leads to the thinning of visual ganglion cells, resulting in ischemia and hypoxia of retinal and choroidal, which may be an important reason for maculopathy in high myopia^37,38^. In addition, DM patients with a duration of over 10 years, fundus lesions gradually aggravated and involved in macular area. Moreover, the patients included in this study often had obvious symptoms, and the prevalence of diabetic maculopathy was higher than that of the community population. Given these reasons, a very high-level prevalence was observed in this study.

In patients with a DR grade < 3, “DR → diabetic maculopathy” was a strong rule, but patients with grades > 3 did not have this strong rule. Studies have shown that for every year an individual has DM, the risk of DR increases 1.29 times^39^. Research conducted by Jeng showed that the risk of non-proliferative DR in cataract surgery patients was 1.48–4.11 times that of the population without cataracts^40^. Our results are similar to those of that study, indicating that the co-occurrence of ocular diseases and hyperglycemia has a certain correlation: one disease will increase the risk of another disease. This may be due to the fact that statins and angiotensin-converting enzyme inhibitors used by cataract patients change the blood-retinal barrier, causing increases in vascular endothelial growth factor, monocyte chemoattractant protein-1, interleukin-1β (IL-1β), and IL-6, thereby increasing the risk of DR^40,41^.

This study has several limitations. First, the participants included only DM patients in the Ophthalmology Department of Henan Provincial People’s Hospital, and the sample size was small. Therefore, its representativeness may be poor, meaning there is a need to further increase the sample size and/or conduct multi-center research. Second, our data are lack of the diagnosis of dyslipidemia and cardiovascular diseases, the impact of these systemic diseases on DR could not be analyzed. Third, as this was a cross-sectional study, it was impossible to judge the sequence and evolution of the various eye diseases, that is, the causality could not be determined. Therefore, in order to reflect the nature of the diseases, prospective studies in a large population are still needed to verify these results.

## Conclusion

The prevalence of DR in DM patients varies with ages, regions, duration of DM and the grade of DR. Patients with one or more eye diseases are at higher risks of developing other eye diseases than those in the general population, and this association rule can be affected by factors such as age, region, duration of the disease, and the severity of DR. This research may provide a valuable scientific basis for understanding the distribution and association rules for the screening, diagnosis, and treatment of DR.

## Data Availability

All relevant data are included in the papers. Contact corresponding author for additional information regarding data access.
